# In This Issue

**DOI:** 10.1002/cfg.450

**Published:** 2004-12


**Comparative and Functional Genomics**
*Comp Funct Genom* 2004; **5**: 571.
Published online in Wiley InterScience (www.interscience.wiley.com). **DOI:** 10.1002/cfg.450

### In this issue

### Differential intron content of maternally and paternally imprinted genes

In their paper, **Marie Fahey *et al.*** describe their analysis of the intron content of human and mouse silenced imprinted genes. They have found that, in both species, there is reduced intron content in maternally (but not paternally) silenced imprinted genes relative to a non-imprinted control set.

### Northern blot reference set for comparison of transcript profiling approaches

**Danielle Kemmer and colleagues** have created a reference data collection of northern blot results and demonstrate how such a collection can be used for a quantitative comparison of expression profiling techniques.

### SOM-based class discovery using ICA-reduced features of microarray data

**Mavroudi *et al.*** have used independent component analysis (ICA) to derive a less redundant representation of complex expression data, prior to applying a SOM-based clustering algorithm. They also use an entropy criterion to allow each gene to be assigned to multiple classes.

### Special section of reviews from the Standards and Ontologies for Functional Genomics Conference 2004

**Midori Harris and Helen Parkinson** introduce this special section with a report on the conference, which brought together computer scientists, ontologists and biomedical scientists working on ontology technology, development and emerging standards for bio-medicine.

**Carole Goble** likens the different communities working on ontologies and the tensions between them to the Montagues and Capulets of Shakespeare's Romeo and Juliet and their conflicts, and proposes constructive ways to achieve harmonious collaborations.

**Susanna Sansone** and colleagues provide an overview of standardization initiatives in the toxicological and environmental sciences, and discuss important issues to be considered if these are to reach their full potential.

**Karen Eilbeck and Suzanna Lewis** provide a step-by-step guide to producing a Sequence Ontology (SO) compliant file of a sequence annotation.

The National Cancer Institute (NCI) thesaurus is a reference terminology covering areas of basic and clinical science with a focus on cancer. **Gilberto Fragoso** and colleagues present an overview of the thesaurus and how to use it.

### Conference Calendar

A listing of genomics related conferences planned for March to May 2005.

Copyright © 2005 John Wiley & Sons, Ltd.

